# Digital model of the cancer cell *–* is this the time for us to debug/reprogram the cancer cell?

**DOI:** 10.3325/cmj.2015.56.578

**Published:** 2015-12

**Authors:** Žarko Vrbica, Marko Jakopović

**Affiliations:** 1 Dubrovnik General Hospital, University of Dubrovnik, Dubrovnik, Croatia *zvrbica@yahoo.com*; 2 Department for Respiratory Diseases Jordanovac, University Hospital Center Zagreb, University of Zagreb Medical School, Zagreb, Croatia

The aim of this article is to present a new view of the cancer cell. In our opinion, mechanical approach to the cancer cell has reached its limits and can no longer help in our struggle to understand and treat cancer. Therefore, there is a need for a new way of looking at the cancer cell and the way it reacts with the environment (1). Our observations are based mainly on experiences with the lung cancer but we think that similar reasoning could be applied to other cancers.

## Biological computer

Our hypothesis is that the human cell should not be analyzed as a biological mechanism but as a biological computer with all the consequences of such an approach. Digitalization enabled us to do things more efficiently and occupying much lesser space than we were able before. Considering the nature's propensity for making things simplier and more efficient, there is no reason to believe that digitalization was not part of the natural development. We are using the power of our computers to analyze different cell reactions, but we still have the mechanistic view of the cell function. Some scientists are even considering the possibility of making biological computers that will be smaller and more efficient than the silicon ones. Actually, it is easy to draw parallels between the human cell and computer systems. The cell is receiving analog inputs through the membrane and cytoplasmic receptors and these receptors are functioning as input units of the computer. After this, the input signals are running through a complex network of molecular interactions working as analog-digital converters until they reach the nucleus, where they interact with the strictly digital DNA system.

The DNA is a code that can be analyzed as a quaternary digital system (with four possible states – bases, as opposed to two bases in electronic binary systems). We are used to have binary digital systems, which are most convenient for electronic based hardware, with only two possible states. However, there is no reason why we should not have digital systems with more than two states. These systems can be even more efficient and compact because they contain more bytes in less space. Scientists dealing with the development of digital systems are very interested in multiple-valued digital systems because of their numerous advantages, like greater speed, greater density of information, better usage of transmission paths, and decreased complexity. The greatest practical interest lies in the application of quaternary multiple valued logic systems, which in theory have the best overall characteristics but in practice there are still problems related to the possibility of their implementation in electronic systems.

**Figure 1:**
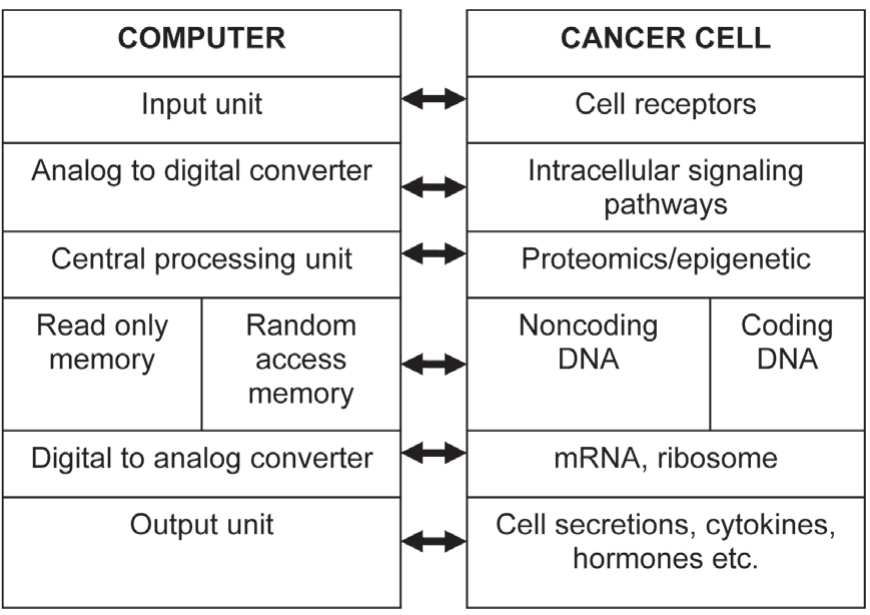
Similarities between computer system and cancer cell.

DNA can be used to perform computational calculations involving storing, retrieving, and processing data and is already applied in nano-biotechnology for engineering bio-molecular systems so that they interact in a fashion that can ultimately result in the computational functionality of a computer (2). A bio-computer consists of a pathway or a series of metabolic pathways involving biological materials that are engineered to behave in a certain manner based upon the conditions (input) imposed on the system. The resulting pathway of reactions constitutes an output, which is based on the engineering design of the bio-computer and can be interpreted as a form of computational analysis. Many models of simple bio-computers have been designed, but their capabilities are still very limited in comparison to commercially available non-bio-computers. Some of these bio-computers have even been used in cancer treatment (3). In our opinion, there is no reason not to accept that the human cell is an advanced bio-computer (Figure 1).

## Data code and program code

Some scientists refer to the coding DNA as a program code, but from the informatics’ point of view this is not a god analogy. Most computer languages make a distinction between programs and other data on which programs operate. The program code is a set of instructions executed directly by the computer's central processing unit. These instructions define what happens to the data that are also digitally coded but are not a program code. If we analyze the cell DNA as a digital system, we can define two distinctive sets of information. The first set are genes, conveying the information about how the basic structural elements should look like. Their counterparts in electronic models are data sets (pictures, spreadsheets etc). The second, more important but yet much less investigated, set is non-coding DNA, which in our model acts as a program code. It makes important decisions and operates on the data sets to make different shapes, forms, and interactions with the environment. If this is so, one should understand why there are not so many differences between the human genome and the genome of some less developed species. Data sets are similar since there is a limited number of available elements that can create different structures. Building blocks are mainly the same but the important difference lies in the program code (building plan). And, actually, the main difference between different species at different developmental stages is the amount of extra-genomic DNA, which is in concordance with our theory. Transposing elements in DNA and epigenetic elements, like non-coding RNA, can act as a type of Boolean logical elements (4) and the whole system is backed-up with the second DNA strand.

## Digital model of the cancer cell and cancer “program code”

Recent studies have focused on understanding the role of the “Dark Genome” – non coding DNA in cancer (5). The importance of non-coding DNA in cancer development is now clear (6) and our hypothesis could explain the reason why non-coding data are so important. The fact that we are dealing with a digital program code that is performing important operations on the cell data and operates in accordance to the input data from the cell environment can have enormous influence on our understanding of the basic causes and principles of cancer development and the way we should approach the treatment.

During the development of a new program, some old programs are usually not deleted but simply inactivated by blocking their execution. If such an old program is activated by mistake it can go awry in the new environment but will still be operational and protect itself from the obstacles in fulfilling its mission. Cancer itself can be caused by an old subroutine activated by mistake or an inherent protective mechanism of the cell in danger from environmental stress, similar to the sporulation of some bacteria. The new evidence of naturally occurring tumors in metazoan organism (7) is advocating this theory.

If the cell is constantly exposed to toxic stimuli, it can eventually start such a program. Every program should be capable of changing not only the way the data are executed but also the data themselves. In this case, at least some mutations in the cancer cell genome could be changes in the data sets made by the program code. Some changes can be made in the living cell but for some changes there is a need to “reset” the system, which can be done only during the cell division. This could explain how different cell types can be found in the same tumor and and how one tumor type can change to another (like NSCLC to the SCLC).

The more developed the program is, the more secure it is from interventions aimed to stop it without the proper code. A program that is developed and adjusted through millennia should be very resistant to such interventions. Contrary to our view of the cancer cell as an invalid cell, it seems that it has a specific function and is able to change in reaction to our treatment interventions and even develop protective mechanisms against some specific substances, at the same time conserving the genetic material and spreading it to distant places in an effort to find a better environment for its survival. This kind of activity could be important in some simple organisms but is detrimental in advanced species.

At least some changes in the cancer cell genome can be explained as deliberate changes made by program code instead as random mutations. Our hypothesis can explain lots of new findings about cancers, like cancer sequence, different type of cancer cells, and even different types of cancers in one tumor and cancer’s development of new mechanisms capable of by-passing our targeted therapies. When we try to mechanically block an important cancer cell mechanism, like we do with targeted therapy, the “cancer program” reacts by finding the way to overcome the obstacle and to continue with the original program, aware of the ongoing attack and acting even more violently to preserve the genetic material. This is what usually happens during standard cancer treatment when the tumor is diminishing at the beginning only to return in a more aggressive form. What we really need to do is to confer to the cancer cell the information that the circumstances have changed and that there is no need for further protective actions. In this case, the digital cell should abort the active program, reset to normal values, or, if this is not possible, activate programmed cell death (the only process in cell biology that contains the word “program” but if we take a deeper look we can see that this process is still defined from the mechanical point of view).

## Implications for further research

The problem with the mechanical approach is that we cannot properly analyze the digital cell. Statistical analysis of different concentrations and numbers of receptors is not applicable to the digital systems. Since the information depends on the sequence of activation of different receptors, it is important to perform time dependent analysis with black box approach to the cancer cell, analyzing the reactions to different inputs in various sequences. The time frame of the sampling is probably the reason why the same experiments obtained different results in different settings.

In our opinion, there has to be a paradigm shift from viewing the cancer cell as a biological mechanism to viewing it as a biological computer. The treatment should not be focused on blocking the mechanisms controlled by program code but to stop the program itself by either dealing directly with the program code or by conveying the information that the program should be stopped. There must be a specific part of the program code (in non-coding DNA) that is active only in cancer cells and we should try to stop that very part without harming the normal cells. If we are dealing with a biological computer running an unknown program, we should use the tools analog to those for analyzing the functions of unknown programs developed for binary computers but we should modify them by having in mind the differences between electronic and biological computers.

Analyzing the cancer cell in digital way could be a cumbersome job, but our opinion is that technology has reached a point when it is able to fulfill this task, only we have to change our point of view. If we can find the key code to stop the cancer cell, it is an effort worth trying. Tailoring research based on that premise with the tools used in analyzing the unknown program code and modified to a biological system could lead us to a better understanding and treatment of cancer.
